# Current State of Connected Sensor Technologies Used During Rehabilitation Care: Protocol for a Scoping Review

**DOI:** 10.2196/60496

**Published:** 2024-10-24

**Authors:** Michelle R Rauzi, Rachael B Akay, Swapna Balakrishnan, Christi Piper, Denise Gobert, Alicia Flach

**Affiliations:** 1 Denver/Seattle Center of Innovation for Veteran-centered and Value Driven Care Rocky Mountain Regional VA Medical Center Aurora, CO United States; 2 Physical Therapy Program Department of Physical Medicine and Rehabilitation University of Colorado Anschutz Medical Campus Aurora, CO United States; 3 Geriatric Research Education and Clinical Center VA Eastern Colorado Health Care System Aurora, CO United States; 4 Interprofessional Health Sciences Ph.D. Program Department of Rehabilitation and Movement Sciences University of Vermont Burlington, VT United States; 5 Strauss Health Sciences Library University of Colorado Anschutz Medical Campus Aurora, CO United States; 6 Department of Physical Therapy College of Health Professions Texas State University Round Rock, TX United States; 7 Exercise Science University of South Carolina Columbia, SC United States

**Keywords:** connected sensor technology, digital health, rehabilitation, rehabilitation care, remote monitoring, telehealth, mHealth, mobile health, wearables, wearable technology

## Abstract

**Background:**

Connected sensor technologies can capture raw data and analyze them using advanced statistical methods such as machine learning or artificial intelligence to generate interpretable behavioral or physiological outcomes. Previous research conducted on connected sensor technologies has focused on design, development, and validation. Published review studies have either summarized general technological solutions to address specific behaviors such as physical activity or focused on remote monitoring solutions in specific patient populations.

**Objective:**

This study aimed to map research that focused on using connected sensor technologies to augment rehabilitation services by informing care decisions.

**Methods:**

The Population, Concept, and Context framework will be used to define inclusion criteria. Relevant articles published between 2008 to the present will be included if (1) the study enrolled adults (population), (2) the intervention used at least one connected sensor technology and involved data transfer to a clinician so that the data could be used to inform the intervention (concept), and (3) the intervention was within the scope of rehabilitation (context). An initial search strategy will be built in Embase; peer reviewed; and then translated to Ovid MEDLINE ALL, Web of Science Core Collection, and CINAHL. Duplicates will be removed prior to screening articles for inclusion. Two independent reviewers will screen articles in 2 stages: title/abstract and full text. Discrepancies will be resolved through group discussion. Data from eligible articles relevant to population, concept, and context will be extracted. Descriptive statistics will be used to report findings, and relevant outcomes will include the type and frequency of connected sensor used and method of data sharing. Additional details will be narratively summarized and displayed in tables and figures. Key partners will review results to enhance interpretation and trustworthiness.

**Results:**

We conducted initial searches to refine the search strategy in February 2024. The results of this scoping review are expected in October 2024.

**Conclusions:**

Results from the scoping review will identify critical areas of inquiry to advance the field of technology-augmented rehabilitation. Results will also support the development of a longitudinal model to support long-term health outcomes.

**Trial Registration:**

Open Science Framework jys53; https://osf.io/jys53

**International Registered Report Identifier (IRRID):**

DERR1-10.2196/60496

## Introduction

Connected sensor technologies are defined as “technology products that process data captured by mobile sensors using algorithms to generate measures of behavioral and/or physiological function” [1,2]. Examples include medical technologies such as ingestibles (smart pills) and dermal patches, along with direct-to-consumer wearables that include activity trackers, heart rate monitors, and smart clothing. Consumer wearables have grown in popularity and ownership over the past decade for a variety of reasons. A 2016 market research survey reported that consumers were motivated to buy wearable technologies to support health, and consumers noted benefits of wearables that include enhancing accountability, improving exercise habits, and becoming more efficient at home and work [3]. These motivations for ownership have stayed consistent over the years. In fact, the American College of Sports Medicine annual survey for fitness trends predicted wearable technology to be the top trend in fitness in the United States for 2024—a trend that has been in the number one spot for 7 of the previous 9 years [4]. This survey also highlighted a new trend for 2024—data-driven training technology—that was defined as using real-time data output such as heart rate to guide workouts [4].

In addition to consumer uptake, health care systems and providers are beginning to adopt connected sensor technologies. A driving factor for health system uptake is the need to deliver health care services to more people at lower costs due to an aging population with high rates of noncommunicable diseases [5]. One approach to reduce costs while promoting better health is through the reallocation of funds to preventative services such as primary care [6] and lifestyle modification programs such as those that promote physical activity. Common limitations of these programs, however, are that maintenance beyond 1 year has not been established [7] and cost-effectiveness is unclear [8]. Alternatively, funds could be used to support remote monitoring programs that use connected sensor technologies with the goal of preventing unnecessary health care utilization. In theory, earlier detection of health deterioration or nonadherence, such as in patients with chronic obstructive pulmonary disease or heart failure, would prompt early, at-home intervention, thus reducing emergency department visits and hospitalizations. While evidence to support such mitigation is mixed [9], one study found that increased adherence was associated with a reduced risk of subsequent hospitalization and death [10], suggesting that a remote monitoring approach may be beneficial for some patients.

Despite increasing popularity among consumers and health care systems, the role of connected sensor technologies in rehabilitation remains unclear. Potential benefits of using patient data from sensor technologies include deeper insights on treatment effectiveness, health behaviors, and symptom patterns [11-13]. However, there may also be negative effects to using such data. Both patients and providers recognize that consumer-grade devices may report inaccurate data [11,12], and they have concerns about data privacy, how to manage and use collected data, and what specific data should be shared [11-13]. Malalignment of patient and provider expectations may contribute to tensions within the therapeutic relationship [14].

As such, there is a need to evaluate how connected sensor technologies can be used effectively to enhance rehabilitation services. The use of connected sensor technologies in rehabilitation practice is relatively new, and much remains unknown given the complexity of available sensor types and heterogeneous patient populations. Most prior studies have focused on the development and validation of connected sensor technologies [15-17] and the infrastructure necessary to support data sharing and cloud computing [18,19]. Previous reviews have focused broadly on technological solutions to address specific behavior interventions such as weight loss [20], while others have focused on tools capable of assessment and remote monitoring of specific patient populations—those with cardiac disease [21], pulmonary disease [22], neurological disorder [23], and cancer [24]. To our knowledge, no review has focused on the use of connected sensor technologies to inform rehabilitation interventions. Such a review would identify the breadth of research, identify knowledge gaps, and elucidate directions for future study while providing clinicians with case examples of how to implement connected sensor technologies into rehabilitation practice.

The aim of this scoping review is to discover and map research that focuses on using connected sensor technologies to augment rehabilitation services by informing care decisions. The general research question guiding this aim is as follows: What is known from existing literature about the remote use of connected sensor technologies integrated into rehabilitation care to monitor patient status and inform care decisions?

## Methods

### Overview

The scoping review will be conducted in accordance with the JBI methodology for scoping reviews [25,26] and reported in line with PRISMA-ScR (Preferred Reporting Items for Systematic Reviews and Meta-Analyses extension for Scoping Reviews) guidelines [27]. We will also follow the 6-stage framework for a scoping review proposed by Arksey and O’Malley [28].

### Stage 1: Identifying the Review Questions

The first stage in the scoping review as outlined by Arksey and O’Malley [28] is to identify specific questions to help guide the search strategy and answer the general question posed above. Thus, we developed the following specific questions:

1. How have researchers studied the use of connected sensor technologies in rehabilitation (eg, what types of studies) as part of a feedback loop between patients and providers?
2. Within the context of rehabilitation care, what types of connected sensor technologies have been used, how were they used, and what types of patient-generated health data were collected?
3. With what patient populations have these connected sensor technologies been used and what has been found?
4. Have researchers explored various partner experiences and perspectives of using connected sensor technologies in rehabilitation, and if so, which partner groups are represented?
5. Based on the scope of research in this area, what are the general limitations and research gaps?

In addition to these questions, we set specific parameters around the research of interest such that each study needed to include (1) a connected sensor technology; (2) data transfer from the patient to the clinician; and (3) use of the data by the clinician to inform the rehabilitation intervention.

### Stage 2: Identifying Relevant Studies

Based on recommendations from JBI, we used the Population, Concept, and Context (PCC) framework to develop initial search terms [26]. These keywords and synonyms will be used to iteratively develop a robust search strategy.

#### Population

The population for the scoping review will be limited to adults (aged ≥18 years). If a study includes both children and adults, the study may be included if data can be separated between the age groups. If data cannot be separated, then the study will be excluded. We will include all patient populations (those with cardiac disease, pulmonary disease, etc). We made this decision because we anticipate that connected sensor technologies used during rehabilitation could be applicable to patient populations other than those studied.

#### Concept

The concept involves studies that include (1) at least one connected sensor technology product that collects patient data; (2) data transfer from the product and patient to the clinician; and (3) use of the data by the clinician to inform the intervention. We used the definition provided in *The Playbook* [1], which defines a connected sensor technology as “products [that] process data captured by mobile sensors using algorithms to generate measures of behavioral and/or physiological function” (p. 79). A connected sensor technology was further defined as one that is capable of electronic transfer; thus, early activity trackers that used an accelerometer to collect step data such as a pedometer would be out of scope for this review as they were not connected technologies. Table 1 shows the different sensor types that will be considered for this review. It is also important to note that multiple sensor types may be combined in one product. Thus, terms such as connected sensor, remote monitoring, accelerometer, Bluetooth, inertial measurement unit, actigraphy, biomedical technology, remote sensing technology, haptic technology, digital technology, and wearable electronic devices will be considered for the search strategy. To capture the concept of data transfer, we will consider terms such as electronic health record integration, cloud, and dashboard. Because the final concept—use of data by the clinician—is difficult to capture using search terms, we will identify this component during full text review.

**Table 1:** Connected sensory technology types^a^ and examples.

Sensor types	Definition	Example product (manufacturer)
Accelerometer	Senses change in person’s (or object’s) linear velocity	CentrePoint Insight Watch (ActiGraph)^b^ Vivoactive 5 (Garmin)^b^
Gyroscope^c^	Senses change in angular velocity	Apple Watch Series 9 (Apple)^b^
Magnetometer	Measures angles relative to the Earth’s magnetic field	MetaMotion C (Mbientla)^d^
Pressure sensor	Measures external pressure	Blue Chip Pressure Mapping Mattress Sensor (Blue Chip Medical) Sensoria Mat Wheelchair Cushion (Sensoria) OpenGO Insoles (Moticon)
IMU^e^	Contains multiple sensors to measure motion in 3 axes; to accomplish this, IMUs include accelerometers, gyroscopes, magnetometers, and pressure sensors	Opal V2R (Clario) Xsens Awinda (Movella) MetaMotion C (Mbientla)^d^
Biomedical sensor	Measures physiological and biological metrics within the human body (heart rate, oxygen saturation, blood glucose, temperature, etc)	Omron Complete 2-in-1 Upper Arm Blood Pressure Monitor and 1-Leak EKG Monitor (Omron)
Temperature sensor	Senses heat and detect changes in temperature	SmartMat (Podimetrics)
Passive infrared sensor	Measures infrared light to detect motion	DomoCare home monitoring system (DomoSafety SA)

^a^This table does not include an exhaustive list of sensor types but rather includes more common examples used in health care.

^b^The CentrePoint Insight Watch, Vivoactive, and Apple Watch also contain a temperature sensor; the Vivoactive and Apple Watch also have biomedical sensors; the Apple Watch uses both an accelerometer and a gyroscope.

^c^Gyroscope and accelerometer are often used concurrently in products.

^d^The MetaMotion C system is an inertial measurement unit that contains a magnetometer in addition to an accelerometer and gyroscope.

^e^IMU: inertial measurement unit.

#### Context

The context will include rehabilitation- and exercise-related studies. As such, the search terms will include rehabilitation, physical therapy, physiotherapy, telehealth, telerehabilitation, digital medicine, and digital health. There will be no other restrictions on the context.

Understanding the context is important to interpret what may be possible in different settings. For example, the Veterans Health Administration (VHA) has been and continues to be a leader in deploying technology solutions for health care. The VHA Telehealth Services program office was established over 20 years ago [29] and has developed necessary infrastructure to support rehabilitation practices that leverage remote patient monitoring to inform care decisions. In contrast, other health care systems may have inadequate infrastructure, which is a significant barrier to organizational implementation of connected sensor technology–augmented services.

#### Search Strategy and Information Sources

A comprehensive literature search was designed and performed by a medical librarian (CP) in February 2024 for the concepts of wearable sensors, rehabilitation, and medical health data. The search was built in Embase and translated to additional databases. Prior to translation, the Embase search strategy was peer-reviewed by a medical librarian following the PRESS (Peer Review of Electronic Search Strategies) guidelines [30]. Relevant publications will be identified by searching the following databases with a combination of standardized index terms and keywords: Ovid MEDLINE ALL (from 1946 to February 22, 2024), Embase (via Elsevier, from 1947 to the present), Web of Science Core Collection (via Thomson Reuters, including Science Citation Index Expanded, from 1974 to the present, and Social Sciences Citation Index, from 1974 to the present), and CINAHL (via EBSCOhost, from 1981 to the present). When possible, the searches will be limited to adult studies, with the publication types of conference abstracts and reviews excluded, and the publication date will be limited to range from 2008 to the present. Multimedia Appendix 1 provides the complete list of database search strategies.

#### Data Management

Following the search, all results will be exported to EndNote 21 (Clarivate Analytics), and duplicates will be initially removed using this reference management software. Results will then be uploaded to Covidence systematic review software (Veritas Health Innovation) for final duplicate removal, screening, and data extraction.

### Stage 3: Article Selection and Eligibility Criteria

This scoping review will consider quantitative, qualitative, and mixed methods study designs for inclusion. While reviews, editorials, and other opinion papers will be excluded, we will manually search such manuscripts for relevant articles. Inclusion and exclusion criteria are summarized in Table 2 according to the PCC framework.

**Figure 1:**
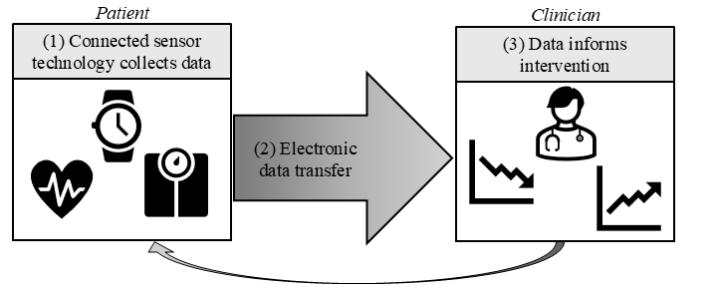
Depiction of the inclusion criteria as defined by the concept domain of the Population, Concept, and Context (PCC) framework.

Studies will be screened for inclusion in 2 stages, and each study will be screened by 2 independent reviewers during both stages. The first stage will involve screening of titles and abstracts; if there are no clear exclusion criteria, the study will advance to the second stage of screening, which is the full-text review. Full-text articles will be retrieved through the University of Colorado library and associated resources, through the Department of Veterans Affairs library, and by contacting corresponding authors. If the full text cannot be found through reasonable means, then the article will be excluded from the review. All inclusion criteria must be met during the full-text review for inclusion in the results, and specifically, each of the 3 steps relevant to the concept domain of the PCC framework must be present (Figure 1). If there are discrepancies at either screening stage, the articles will be discussed as a team. The results of the search will be reported in full in the final scoping review and presented in a PRISMA (Preferred Reporting Items for Systematic Reviews and Meta-Analyses) flow diagram [31].

**Table 2:** Inclusion and exclusion criteria.

Domain	Inclusion	Exclusion
Population	Adults (aged ≥18 years)	Sample aged <18 years Does not involve human subjects (animals, etc)
Concept	Connected sensor technology product used in the study either as the primary or secondary technology (eg, secondary to virtual reality intervention) Collects patient data primarily through passive means Data transfer from the participant’s connected sensor technology to a clinician The clinician uses the data to inform an intervention	Study not related to an intervention (observational, validation, or reliability study; review; opinion; etc.) No data transfer or data transfer requires ongoing active input from the participant (eg, paper or electronic diary) The data are not used to inform the intervention (eg, accelerometry data collected only for study outcome)
Context	Intervention is relevant to rehabilitation (exercise, physical activity, symptom management, etc)	Intervention is not relevant to rehabilitation (eg, medication adherence)
Other	—a	Full text not available in English

^a^Not applicable.

### Stage 4: Data Extraction and Charting

We will pilot a custom data extraction table within Covidence and/or Excel (Microsoft) before starting formal data extraction. Data extraction will include article characteristics (eg, authors, title, journal, and year of publication), study characteristics (study design, country where study was conducted, aim or aims, population studied, etc), and data pertinent to the scoping review questions. As we are interested in describing what is known about connected sensor technologies integrated into rehabilitation interventions, we will extract technology and data characteristics (eg, type of connected sensor technology, name and manufacturer, prior validation, and type of data collected from the connected sensor technology). In addition, we will describe the method(s) used for data transfer and how the data were used to inform the intervention. The data extraction table will be updated as we become more familiar with the literature and identify salient data to answer the research questions. Further details of planned data extraction are in Table 3.

Primary data extraction will be completed by MRR and checked by a second member of the team for completeness. Any discrepancies that arise will be discussed as a team for resolution. Authors of papers will be contacted when needed to request missing or additional data. Modifications to data extraction will be detailed in the full scoping review.

**Table 3:** Data extraction plan.

Category	Data extracted
Population	Sample size Participant demographics (sex, age, etc) Population diagnoses (chronic obstructive pulmonary disease, congestive heart failure, Parkinson disease, etc)
Concept	Type of sensor technology Type of data collected from sensor technology (heart rate, step count, sleep duration, etc) Method of data collection Data transfer methods Compatibility (platform and operating system) How sensor technology was used Prior validation Method of validation (if applicable) Co-occurring digital health technologies (eg, artificial intelligence)
Context	Health care system Type of health care setting (hospital, clinic, home, etc) Type of rehabilitation (physical therapy, occupational therapy, etc) Country

### Stage 5: Data Synthesis

The scope of the literature will be described using counts (n), percentages, means, medians, and measures of variance as appropriate. For example, we will describe the number and type (randomized controlled trial, quasi-experimental, etc) of studies that have been conducted. We will also describe the number of connected sensory technologies studied by category (accelerometer, magnetometer, etc) and the populations in which the products were studied (those musculoskeletal or neurological disorders, healthy adults, etc). These results will be presented in figures and tables. A narrative approach will be used to describe how connected sensor technologies were used during rehabilitation interventions and to summarize any qualitative data that may be included in the review.

### Stage 6: Partner Engagement

Once results are synthesized, we will present them to various partner groups including patients and rehabilitation clinicians. Partner feedback will be incorporated into the results and discussion to afford a broader presentation and interpretation of the findings of the scoping review. We anticipate that partner input will help identify areas that have been understudied or not studied, situate the results in the context of priorities of various partner groups, and identify salient future directions for research.

## Results

We conducted initial searches to refine the search strategy in February 2024. Through this process, we added a date requirement to include studies from 2008 to the present. We aim to complete the scoping review through data synthesis (stage 5) by October 2024, followed by partner engagement (stage 6) before dissemination in a scientific journal.

## Discussion

### Principal Findings

The findings from this scoping review will identify how connected sensor technologies have been integrated into rehabilitation care and with which populations. We suspect that many of the interventions using connected sensor technologies will focus on behavior change and that the most common type of connected sensor technology will be consumer activity monitors. We also anticipate that connected sensor technologies will be commonly applied to cardiac and pulmonary rehabilitation programs, because these programs are well established [32-34] and much work has been done to evaluate the effectiveness of telehealth delivery for these programs [32,35,36].

### Comparison to Prior Work

Previous reviews have evaluated similar concepts to the ones described in this manuscript. For example, Wei and Wu [37] conducted a review of preclinical studies that evaluated the ability of sensors to assess, recognize, and classify human movements in patient populations. Similarly, Kim et al [38] reviewed the use of sensors for upper-extremity assessment and intervention in a population with stroke. Only 5 of the reviewed studies used the sensor data to assist with an intervention, but none of the technologies used in the studies transferred data. As a result, the clinician was not actively involved in using the sensor data to inform treatments. Another review focused on the use of wearable technologies in oncology populations and found 10 studies that used wearables to effect health outcomes [39]; however, there were no details about the interventions and how or if sensor data were used by the clinician. The review described in this protocol will address these limitations.

### Strengths and Limitations

The scoping review protocol has strengths that will improve the reliability of its findings. First, a strength of this review is the use of established and rigorous methodology [25,26,28]. Second, we are a diverse team with different research backgrounds and interests in technological applications; this diversity will enhance the findings by accounting for various perspectives. Additionally, we will use step 6 (partner engagement) of the Arksey and O’Malley [28] framework, which will improve the interpretation, communication, and dissemination of the findings.

The scoping review also has limitations. First, there is a lack of standard language to describe the data transfer process and how sensor data are used by a clinician; therefore, our search may miss articles that use terms not captured in our search strategy. This limitation also highlights the need for future work to generate consensus for such terminology. Another limitation is that the search strategy may miss eligible articles in which a connected sensor technology was used but was secondary to a different health technology (eg, virtual reality intervention that included connected sensor technologies). Similarly, our search may miss technologies that were studied privately or went directly to consumer markets.

### Dissemination Plan

Findings from this scoping review will be disseminated through various communication channels. First, we will share our findings with partners through presentations and discussions. Then, formal findings will be disseminated in peer-reviewed journals, presented at scientific conferences, and shared on social medial platforms such as LinkedIn.

### Conclusion

We designed this scoping review to help map how connected sensor technologies have been studied to augment rehabilitation interventions. Results from the scoping review will identify the current state of this research while highlighting critical areas of inquiry to advance this field. Results will also support the development of a longitudinal care model to prioritize long-term health outcomes.

## Appendix

Multimedia Appendix 1 Search strategy.

## Abbreviations

PCC: Population, Concept, and Context
PRESS: Peer Review of Electronic Search Strategies
PRISMA: Preferred Reporting Items for Systematic Reviews and Meta-Analyses
PRISMA-ScR: Preferred Reporting Items for Systematic Reviews and Meta-Analyses extension for Scoping Reviews
VHA: Veterans Health Administration

## Data Availability

Data sharing is not applicable to this protocol as no datasets were generated or analyzed.
