# Supplementary material for: Current State of Connected Sensor Technologies Used During Rehabilitation Care: Protocol for a Scoping Review
Source: JMIR Res Protoc. 2024 Oct 24;13:e60496. doi: 10.2196/60496 (PMC11544342; doi:10.2196/60496)
Supplement: Multimedia Appendix 1 [file resprot_v13i1e60496_app1.docx]

# Multimedia Appendix 1: Search strategy

| **Date** | 23-Feb-24 |
| --- | --- |
|  |  |
| **Databases** |  |
| Ovid Medline | 673 |
| Embase | 805 |
| CINAHL | 263 |
| Web of Science | 1029 |
| Total | 2770 |
| Duplicates removed in EndNote | 1025 |
| Duplicates removed in Covidence | 0 |
| Unique articles to review | 1745 |

| **Embase (via Elsevier, Embase.com, 1947 to present)** | |  |
| --- | --- | --- |
| Search date: February 23, 2024 | |  |
| No. | Query | Results |
| #50 | #49 AND [2008-2025]/py | 805 |
| #49 | #48 NOT ('conference abstract':it OR 'review':it) | 837 |
| #48 | #47 NOT ('juvenile'/exp NOT 'adult'/exp) | 1466 |
| #47 | #22 AND #37 AND #46 | 1534 |
| #46 | #38 OR #39 OR #40 OR #41 OR #42 OR #43 OR #44 OR #45 | 425369 |
| #45 | (cloud NEAR/3 (process* OR service* OR storage* OR comput*)):ti,ab | 4424 |
| #44 | 'cloud computing'/de | 3753 |
| #43 | (((distant OR remote OR tele) NEAR/3 (monitor* OR surveillanc*)):ti,ab) OR telesurveillanc*:ti,ab OR telemonitor*:ti,ab | 14056 |
| #42 | 'telemonitoring'/de | 6072 |
| #41 | ('closed loop' NEAR/3 (feedback OR stimulation* OR system* OR design* OR device* OR interface*)):ti,ab | 7873 |
| #40 | 'application programming interface'/exp OR 'application programming interface*':ti,ab | 1358 |
| #39 | ((health OR medical OR patient OR provider) NEAR/2 (record* OR dashboard* OR integration*)):ti,ab | 356830 |
| #38 | 'electronic health record'/de OR 'electronic medical record'/de OR 'electronic patient record'/de | 131419 |
| #37 | #23 OR #24 OR #25 OR #26 OR #27 OR #28 OR #29 OR #30 OR #31 OR #32 OR #33 OR #34 OR #35 OR #36 | 1973460 |
| #36 | rehab*:ti,ab OR telerehab*:ti,ab OR 'tele-rehab*':ti,ab | 315104 |
| #35 | 'rehabilitation'/exp | 515086 |
| #34 | (recreation* NEAR/3 therap*):ti,ab | 1013 |
| #33 | 'recreational therapy'/de | 713 |
| #32 | (functional NEAR/3 (training* OR assessment* OR activit* OR task* OR mobilit* OR rehab*)):ti,ab | 98879 |
| #31 | 'functional assessment'/de OR 'fuctional training' | 72746 |
| #30 | kinesi$therap*:ti,ab OR yoga:ti,ab OR 'tai chi':ti,ab OR 'tai ji':ti,ab OR 'taiji quan':ti,ab OR taijiquan:ti,ab | 14723 |
| #29 | 'kinesiotherapy'/exp OR 'exercise'/exp | 513441 |
| #28 | 'physical activit*':ti,ab OR walking:ti,ab OR cycling:ti,ab OR biking:ti,ab OR swimming:ti,ab OR 'aquatic therap*':ti,ab OR 'weight lifting':ti,ab OR weightlift*:ti,ab OR powerlift*:ti,ab OR 'power lifting':ti,ab OR exercis*:ti,ab | 859958 |
| #27 | 'physical activity'/exp | 554963 |
| #26 | 'ergotherap*':ti,ab OR 'occupational therap*':ti,ab OR 'task specific activit*':ti,ab | 27219 |
| #25 | 'occupational therapy'/exp | 29718 |
| #24 | 'physical therap*':ti,ab OR 'physio therap*':ti,ab OR physiotherap*:ti,ab OR 'rehabilitation therap*':ti,ab | 107064 |
| #23 | 'physiotherapy'/exp | 117596 |
| #22 | #1 OR #2 OR #3 OR #4 OR #5 OR #6 OR #7 OR #8 OR #9 OR #10 OR #11 OR #12 OR #13 OR #14 OR #15 OR #16 OR #17 OR #18 OR #19 OR #20 OR #21 | 259016 |
| #21 | 'electric* myogram*':ti,ab OR 'electro myogram*':ti,ab OR 'emg':ti,ab OR 'electromyogram*':ti,ab | 59223 |
| #20 | 'electromyogram'/de | 32678 |
| #19 | biometric*:ti,ab OR biometry:ti,ab OR biomets:ti,ab OR 'digital biomarker*':ti,ab | 20616 |
| #18 | 'biometry'/de | 23514 |
| #17 | (((hearing OR listening) NEAR/2 (aid* OR device* OR apparatus)):ti,ab) OR ((auditory NEAR/2 (appliance* OR prosthe*)):ti,ab) | 15280 |
| #16 | 'hearing aid'/exp | 36662 |
| #15 | (('internet of things' OR iot) NEAR/3 (sensor* OR medical* OR medicine*)):ti,ab | 918 |
| #14 | 'internet of things'/de AND ('sensor'/exp OR 'medicine'/exp) | 1588 |
| #13 | magnetometer*:ti,ab OR 'magneto meter*':ti,ab OR sentimag*:ti,ab | 4018 |
| #12 | 'magnetometer'/de | 3148 |
| #11 | 'actimetry'/de | 12533 |
| #10 | (((imu OR inertia*) NEAR/3 sensor*):ti,ab) OR ((inertia* NEAR/2 (measure* OR model) NEAR/2 (unit* OR device* OR sensor*)):ti,ab) | 6439 |
| #9 | 'inertial sensor'/de | 2002 |
| #8 | ((activity OR fitness) NEAR/3 (tracker* OR monitor*)):ti,ab | 27606 |
| #7 | 'activity tracker'/exp | 5289 |
| #6 | '3dnx':ti,ab OR accelerometer*:ti,ab OR actical:ti,ab OR adxl322:ti,ab OR 'dynaport minimod':ti,ab OR 'genea':ti,ab OR 'gt1m':ti,ab OR 'gt3x':ti,ab OR minimod:ti,ab | 25045 |
| #5 | 'accelerometer'/exp OR 'accelerometry'/de | 26767 |
| #4 | ((instrumented NEAR/2 (cloth* OR insole*)):ti,ab) OR 'flexible sensor*':ti,ab OR ((textile* NEAR/2 electronic*):ti,ab) OR 'embedded technolog*':ti,ab | 1362 |
| #3 | (smart NEAR/2 (watch* OR cloth* OR vest* OR suit* OR pant* OR textile* OR insole* OR sock* OR headband* OR 'head band*' OR cap OR hat* OR headphone* OR 'head phone*' OR band OR monitor* OR earring* OR textile* OR wristband* OR 'mouth guard*' OR collar* OR sleeve* OR garment* OR device* OR glasses OR shoe*)):ti,ab | 4665 |
| #2 | ((wearable NEAR/2 (apparatus OR equipment* OR device* OR monitor* OR sensor* OR technolog*)):ti,ab) OR wearable*:ti,ab | 30053 |
| #1 | 'wearable device'/exp | 13957 |

| **MEDLINE (via Ovid MEDLINE® ALL, 1946 to February 22, 2024)** | |  |
| --- | --- | --- |
| Search date: February 23, 2024 | |  |
| 1 | exp "wearable electronic devices"/ | 19811 |
| 2 | ((wearable ADJ2 (apparatus OR equipment* OR device* OR monitor* OR sensor* OR technolog*)) OR wearable*).tw,kf | 29947 |
| 3 | (smart ADJ2 (watch* OR cloth* OR vest* OR suit* OR pant* OR textile* OR insole* OR sock* OR headband* OR "head band*" OR cap OR hat* OR headphone* OR "head phone*" OR monitor* OR earring* OR band OR textile* OR wristband* OR "mouth guard*" OR collar* OR sleeve* OR garment* OR device OR glasses OR shoe*)).tw,kf | 2812 |
| 4 | ((instrumented ADJ2 (cloth* OR insole*)) OR "flexible sensor*" OR (textile* ADJ2 electronic*) OR "embedded technolog*").tw,kf | 1790 |
| 5 | exp "Accelerometry"/ | 12412 |
| 6 | ("3dnx" OR accelerometer* OR actical OR adxl322 OR "dynaport minimod" OR "genea" OR "gt1m" OR "gt3x" OR minimod).tw,kf | 20584 |
| 7 | "Fitness Trackers"/ | 1158 |
| 8 | (((activity OR fitness) ADJ3 (tracker* OR monitor*)).tw,kf) OR "fitness tracker".tw,kf OR "fitness trackers".tw,kf OR "activity tracker".tw,kf | 20622 |
| 9 | (((IMU OR inertia*) ADJ3 sensor*) OR (inertia* ADJ2 (measure* OR model) ADJ2 (unit* OR Device* OR sensor*))).tw,kf | 6146 |
| 10 | "actigraphy"/ | 4832 |
| 11 | (magnetometer* OR "magneto meter*" OR sentimag*).tw,kf | 3925 |
| 12 | ("internet of things"/ AND exp medicine/) | 75 |
| 13 | (("internet of things" OR IOT) ADJ3 (sensor* OR medical* OR medicine*)).tw,kf | 960 |
| 14 | exp "hearing aids"/ | 22233 |
| 15 | (((hearing OR listening) ADJ2 (aid* OR device* OR apparatus)) OR (auditory ADJ2 (appliance* OR prosthe*))).tw,kf | 13398 |
| 16 | "biometry"/ | 28947 |
| 17 | (biometric* OR biometry OR biomets OR "digital biomarker*").tw,kf | 15829 |
| 18 | "Electromyography"/ | 85461 |
| 19 | ("electric* myogram*" OR "electro myogram*" OR "EMG" OR "electromyogram*").tw,kf | 42641 |
| 20 | or/1-19 | 247155 |
| 21 | exp "Physical Therapy Modalities"/ OR "Physical Therapy Specialty"/ | 184686 |
| 22 | ("physical therap*" OR "physio therap*" OR physiotherap* OR "rehabilitation therap*").tw,kf | 70145 |
| 23 | "occupational therapy"/ | 15179 |
| 24 | ("ergotherap*" OR "occupational therap*" OR "task specific activit*").ti,ab | 17712 |
| 25 | exp "Exercise"/ | 253652 |
| 26 | ("physical activit*" OR "walking" OR "cycling" OR "biking" OR "swimming" OR "aquatic therap*" OR "weight lifting" OR "weightlift*" OR "powerlift*" OR "power lifting" OR exercise*).ti,ab | 653199 |
| 27 | (exercising OR kinesi?therap* OR yoga OR "tai chi" OR "tai ji" OR "taiji quan" OR taijiquan).ti,ab | 22971 |
| 28 | (functional ADJ3 (training* OR assessment* OR activit* OR task* OR mobilit* OR rehab*)).tw,kf | 71217 |
| 29 | "Recreation therapy"/ | 143 |
| 30 | (recreation* ADJ3 therap*).tw,kf | 692 |
| 31 | **exp "rehabilitation"/** | 359753 |
| 32 | (rehab* OR telerehab* OR "e rehab*" OR "tele-rehab*").tw,kf | 233356 |
| 33 | or/21-32 | 1228313 |
| 34 | exp "electronic health records"/ | 28848 |
| 35 | ((health OR medical OR patient OR provider) ADJ2 (record* OR dashboard* OR integration*)).tw,kf | 214219 |
| 36 | "application programming interface*".tw,kf | 1235 |
| 37 | ("closed loop" ADJ3 (feedback OR stimulation* OR system* OR design* OR device* OR interface*)).tw,kf | 5977 |
| 38 | (((distant OR remote OR tele) ADJ3 (monitor* OR surveillanc*)) OR telesurveillanc* OR telemonitor*).tw,kf | 9911 |
| 39 | exp "cloud computing"/ | 1378 |
| 40 | (cloud ADJ3 (process* OR service* OR storage* OR comput*)).tw,kf | 3816 |
| 41 | or/34-40 | 244300 |
| 42 | 20 AND 33 AND 41 | 822 |
| 43 | 42 NOT (Adolescent/ NOT exp Adult/) | 8011 |
| 44 | 43 NOT review.pt | 709 |
| 45 | limit 44 to yr=2008-current | 673 |

| **CINAHL (Cumulative Index to Nursing and Allied Health Literature via EBSCOhost, 1981 to present)** | | | |
| --- | --- | --- | --- |
| Search date: February 23, 2024 | | | |
| **#** | **Query** | **Limiters/Expanders** | **Results** |
| S29 | S28 AND PY 2008-2025 | Expanders - Apply equivalent subjects Search modes - Boolean/Phrase | 263 |
| S28 | S27 NOT (MH Adolescent+ NOT (MH Adult OR MH Aged+ OR MH “Middle Age”)) | Expanders - Apply equivalent subjects Search modes - Boolean/Phrase | 290 |
| S27 | S12 AND S20 AND S26 | Expanders - Apply equivalent subjects Search modes - Boolean/Phrase | 290 |
| S26 | S21 OR S22 OR S23 OR S24 OR S25 | Expanders - Apply equivalent subjects Search modes - Boolean/Phrase | 146,858 |
| S25 | TX(cloud N3 (process* OR service* OR storage* OR comput*)) | Expanders - Apply equivalent subjects Search modes - Boolean/Phrase | 1,597 |
| S24 | TX(((distant OR remote OR tele) N3 (monitor* OR surveillanc*)) OR telesurveillanc* OR telemonitor*) | Expanders - Apply equivalent subjects Search modes - Boolean/Phrase | 3,577 |
| S23 | TX("closed loop" N3 (feedback OR stimulation* OR system* OR design* OR device* OR interface*)) | Expanders - Apply equivalent subjects Search modes - Boolean/Phrase | 930 |
| S22 | TX( "application programming interface*") | Expanders - Apply equivalent subjects Search modes - Boolean/Phrase | 209 |
| S21 | TX((health OR medical OR patient OR provider) N2 (record* OR dashboard* OR integration*)) | Expanders - Apply equivalent subjects Search modes - Boolean/Phrase | 141,138 |
| S20 | S13 OR S14 OR S15 OR S16 OR S17 OR S18 OR S19 | Expanders - Apply equivalent subjects Search modes - Boolean/Phrase | 403,011 |
| S19 | TI(rehab* OR telerehab* OR "e rehab*" OR "tele-rehab*") OR AB(rehab* OR telerehab* OR "e rehab*" OR "tele-rehab*") | Expanders - Apply equivalent subjects Search modes - Boolean/Phrase | 113,725 |
| S18 | TI(recreation* N3 therap*) OR AB(recreation* N3 therap*) | Expanders - Apply equivalent subjects Search modes - Boolean/Phrase | 1,214 |
| S17 | TI(functional N3 (training* OR assessment* OR activit* OR task* OR mobilit* OR rehab*)) OR AB(functional N3 (training* OR assessment* OR activit* OR task* OR mobilit* OR rehab*)) | Expanders - Apply equivalent subjects Search modes - Boolean/Phrase | 22,981 |
| S16 | TI(exercise OR exercising OR kinesi?therap* OR yoga OR "tai chi" OR "tai ji" OR "taiji quan" OR taijiquan) OR AB(exercise OR exercising OR kinesi?therap* OR yoga OR "tai chi" OR "tai ji" OR "taiji quan" OR taijiquan) | Expanders - Apply equivalent subjects Search modes - Boolean/Phrase | 142,566 |
| S15 | TI("physical activit*" OR walking OR cycling OR biking OR swimming OR "aquatic therap*" OR "weight lifting" OR weightlift* OR powerlift* OR "power lifting" OR exercise*) OR AB("physical activit*" OR walking OR cycling OR biking OR swimming OR "aquatic therap*" OR "weight lifting" OR weightlift* OR powerlift* OR "power lifting" OR exercise*) | Expanders - Apply equivalent subjects Search modes - Boolean/Phrase | 234,208 |
| S14 | TI(ergotherap* OR "occupational therap*" OR "task specific activit*") OR AB(ergotherap* OR "occupational therap*" OR "task specific activit*") | Expanders - Apply equivalent subjects Search modes - Boolean/Phrase | 27,270 |
| S13 | TI("physical therap*" OR "physio therap*" OR physiotherap* OR "rehabilitation therap*") OR AB("physical therap*" OR "physio therap*" OR physiotherap* OR "rehabilitation therap*") | Expanders - Apply equivalent subjects Search modes - Boolean/Phrase | 48,731 |
| S12 | S1 OR S2 OR S3 OR S4 OR S5 OR S6 OR S7 OR S8 OR S9 OR S10 OR S11 | Expanders - Apply equivalent subjects Search modes - Boolean/Phrase | 57,186 |
| S11 | TX("electric* myogram*" OR "electro myogram*" OR "EMG" OR "electromyogram*") | Expanders - Apply equivalent subjects Search modes - Boolean/Phrase | 8,770 |
| S10 | TX(biometric* OR biometry OR biomets OR "digital biomarker*") | Expanders - Apply equivalent subjects Search modes - Boolean/Phrase | 13,302 |
| S9 | TX(((hearing OR listening) N2 (aid* OR device* OR apparatus)) OR (auditory N2 (appliance* OR prosthe*))) | Expanders - Apply equivalent subjects Search modes - Boolean/Phrase | 13,898 |
| S8 | TX(("internet of things" OR IOT) N3 (sensor* OR medical* OR medicine*)) | Expanders - Apply equivalent subjects Search modes - Boolean/Phrase | 76 |
| S7 | TX(magnetometer* OR "magneto meter*" OR sentimag*) | Expanders - Apply equivalent subjects Search modes - Boolean/Phrase | 130 |
| S6 | TX(((IMU OR inertia*) N3 sensor*) OR (inertia* N2 (measure* OR model) N2 (unit* OR Device* OR sensor*))) | Expanders - Apply equivalent subjects Search modes - Boolean/Phrase | 1,254 |
| S5 | TX(((activity OR fitness) N3 (tracker* OR monitor*)) OR "fitness tracker" OR "fitness trackers" OR "activity tracker") | Expanders - Apply equivalent subjects Search modes - Boolean/Phrase | 5,977 |
| S4 | TX(3dnx OR accelerometer* OR actical OR adxl322 OR "dynaport minimod" OR genea OR gt1m OR gt3x OR minimod) | Expanders - Apply equivalent subjects Search modes - Boolean/Phrase | 8,849 |
| S3 | TX((instrumented N2 (cloth* OR insole*)) OR "flexible sensor*" OR (textile* N2 electronic*) OR "embedded technolog*") | Expanders - Apply equivalent subjects Search modes - Boolean/Phrase | 68 |
| S2 | TX(smart N2 (watch* OR cloth* OR vest* OR suit* OR pant* OR textile* OR insole* OR sock* OR headband* OR "head band*" OR cap OR hat* OR headphone* OR "head phone*" OR monitor* OR earring* OR band OR textile* OR wristband* OR "mouth guard*" OR collar* OR sleeve* OR garment* OR device OR glasses OR shoe*)) | Expanders - Apply equivalent subjects Search modes - Boolean/Phrase | 956 |
| S1 | TX((wearable N2 (apparatus OR equipment* OR device* OR monitor* OR sensor* OR technolog*)) OR wearable*) | Expanders - Apply equivalent subjects Search modes - Boolean/Phrase | 7,141 |

| **Web of Science Core Collection (via Clarivate Analytics, including Science Citation Index Expanded and Social Sciences Citation Index, 1974 to February 20, 2024)** | | |
| --- | --- | --- |
| Search date: February 24, 2024 | |  |
| # | Search Query | Results |
| 1 | TS=((wearable NEAR/2 (apparatus OR equipment* OR device* OR monitor* OR sensor* OR technolog*)) OR wearable*) | 54765 |
| 2 | TS=(smart NEAR/2 (watch* OR cloth* OR vest* OR suit* OR pant* OR textile* OR insole* OR sock* OR headband* OR "head band*" OR cap OR hat* OR headphone* OR "head phone*" OR monitor* OR earring* OR band OR textile* OR wristband* OR "mouth guard*" OR collar* OR sleeve* OR garment* OR device OR glasses OR shoe*)) | 17754 |
| 3 | TS=((instrumented NEAR/2 (cloth* OR insole*)) OR "flexible sensor*" OR (textile* NEAR/2 electronic*) OR "embedded technolog*") | 5248 |
| 4 | TS=("3dnx" OR accelerometer* OR actical OR adxl322 OR "dynaport minimod" OR "genea" OR "gt1m" OR "gt3x" OR minimod) | 34410 |
| 5 | TS=(((activity OR fitness) NEAR/3 (tracker* OR monitor*)) OR "fitness tracker" OR "fitness trackers" OR "activity tracker") | 34665 |
| 6 | TS=(((IMU OR inertia*) NEAR/3 sensor*) OR (inertia* NEAR/2 (measure* OR model) NEAR/2 (unit* OR Device* OR sensor*))) | 12329 |
| 7 | TS=(magnetometer* OR "magneto meter*" OR sentimag*) | 27651 |
| 8 | TS=(("internet of things" OR IOT) NEAR/3 (sensor* OR medical* OR medicine*)) | 5637 |
| 9 | TS=(((hearing OR listening) NEAR/2 (aid* OR device* OR apparatus)) OR (auditory NEAR/2 (appliance* OR prosthe*))) | 11985 |
| 10 | TS=(biometric* OR biometry OR biomets OR "digital biomarker*") | 30142 |
| 11 | TS=("electric* myogram*" OR "electro myogram*" OR "EMG" OR "electromyogram*") | 43030 |
| 12 | #1 OR #2 OR #3 OR #4 OR #5 OR #6 OR #7 OR #8 OR #9 OR #10 OR #11 | 254701 |
| 13 | TS=("physical therap*" OR "physio therap*" OR physiotherap* OR "rehabilitation therap*") | 58872 |
| 14 | TS=(ergotherap* OR "occupational therap*" OR "task specific activit*") | 18012 |
| 15 | TS=("physical activit*" OR walking OR cycling OR biking OR swimming OR "aquatic therap*" OR "weight lifting" OR weightlift* OR powerlift* OR "power lifting" OR exercise*) | 2419706 |
| 16 | TS=(exercise OR exercising OR kinesi$therap* OR yoga OR "tai chi" OR "tai ji" OR "taiji quan" OR taijiquan) | 512672 |
| 17 | TS=(functional NEAR/3 (training* OR assessment* OR activit* OR task* OR mobilit* OR rehab*)) | 81089 |
| 18 | TS=(recreation* NEAR/3 therap*) | 643 |
| 19 | TS=(rehab* OR telerehab* OR "e rehab*" OR "tele-rehab*") | 249815 |
| 20 | #13 OR #14 OR #15 OR #16 OR #17 OR #18 OR #19 | 2723661 |
| 21 | TS=((health OR medical OR patient OR provider) NEAR/2 (record* OR dashboard* OR integration*)) | 233657 |
| 22 | TS=("application programming interface*") | 2818 |
| 23 | TS=("closed loop" NEAR/3 (feedback OR stimulation* OR system* OR design* OR device* OR interface*)) | 35126 |
| 24 | TS=(((distant OR remote OR tele) NEAR/3 (monitor* OR surveillanc*)) OR telesurveillanc* OR telemonitor*) | 17364 |
| 25 | TS=(cloud NEAR/3 (process* OR service* OR storage* OR comput*)) | 41644 |
| 26 | #21 OR #22 OR #23 OR #24 OR #25 | 329001 |
| 27 | #12 AND #20 AND #26 | 1073 |
| 28 | #12 AND #20 AND #26 Timespan: 2008-01-01 to 2025-01-01 | 1029 |
